# Investigation of *Sheng Mai Yin* in the treatment of anthracycline-induced frequent premature ventricular contractions in cancer patients: study protocol for a multicenter, randomized, double-blind, placebo-controlled clinical trial

**DOI:** 10.3389/fcvm.2025.1670053

**Published:** 2025-11-24

**Authors:** Qiwen Yang, Chaofeng Niu, Meng Li, Tangshun Wang, Ce Wang, Longzhen Han, Lan Wei, Yayue Zhang, Dong Li, Shuhua Yi, Duolao Wang, Lijing Zhang

**Affiliations:** 1 Department of Cardiology, Dongzhimen Hospital, Beijing University of Chinese Medicine, Beijing, China; 2 Liverpool Centre for Cardiovascular Science at University of Liverpool, Liverpool John Moores University, and Liverpool Heart & Chest Hospital, Liverpool, United Kingdom; 3 Department of Surgery, Dongzhimen Hospital, Beijing University of Chinese Medicine, Beijing, China; 4 Department of Oncology and Hematology, Dongzhimen Hospital, Beijing University of Chinese Medicine, Beijing, China; 5 Department of Traditional Chinese Medicine, Peking University Third Hospital, Beijing, China; 6 Institute of Hematology and Blood Disease Hospital, Chinese Academy of Medical Sciences, Peking Union Medical College, Tianjin, China; 7 Department of Clinical Sciences, Liverpool School of Tropical Medicine, Liverpool, United Kingdom

**Keywords:** traditional Chinese medicine, *Sheng Mai Yin*, *Qi* and *Yin* deficiency syndrome, anthracycline-induced cardiotoxicity, frequent premature ventricular contractions, cardio-oncology

## Abstract

**Introduction:**

Anthracycline-induced cardiotoxicity is a major concern in cancer treatment, as it can lead to various arrhythmias, with frequent premature ventricular contractions (PVCs) being one of the most common types. *Sheng Mai Yin* (SMY), a widely used Chinese herbal compound in China, has shown potential in treating anthracycline-induced cardiac dysfunction and arrhythmias. However, the evidence supporting its efficacy is limited due to methodological flaws in prior studies. Therefore, high-quality trials are essential to rigorously evaluate the efficacy and safety of SMY.

**Methods:**

This multicenter, randomized, double-blind, placebo-controlled trial will assess the efficacy and safety of SMY in treating frequent PVCs induced by anthracycline chemotherapy. A total of 212 patients with breast cancer or malignant lymphoma undergoing anthracycline-based chemotherapy, who have been diagnosed with new-onset frequent PVCs and *Qi* and *Yin* deficiency syndrome, will be enrolled. Participants will be randomly assigned to receive either SMY or a placebo for 8 weeks, alongside standard medications. The primary outcome is the reduction rate in PVC frequency. Secondary outcomes include PVC symptom scores, Traditional Chinese Medicine syndrome scores, cardiac dysfunction biomarkers, and major adverse cardiovascular events.

**Discussion:**

The results of this trial are expected to provide robust evidence regarding the efficacy and safety of SMY in the treatment of anthracycline-induced frequent PVCs.

**Trial registration:**

http://itmctr.ccebtcm.org.cn. Registration number: ITMCTR2024000858.

## 1 Introduction

Since the 1990s, advancements in cancer diagnosis and treatment have significantly prolonged the survival of cancer patients, making cardiovascular disease (CVD) the leading cause of mortality (1, 2). Consequently, increasing attention has been directed toward the cardiovascular adverse effects associated with cancer therapies. Anthracyclines, a key component of many first-line chemotherapy regimens, exhibit a well-documented dose-dependent risk of anthracycline-induced cardiotoxicity (ACT) (3, 4). ACT often manifests as cardiac dysfunction (3%–48%) and arrhythmias (10%–65.5%), posing a significant threat to patients' quality of life and prognosis (5–7). Premature ventricular contractions (PVCs) are common in the general population (8, 9). Asymptomatic or mildly symptomatic PVCs with low burden and preserved ventricular function are generally considered benign. However, frequent PVCs have been associated with an increased risk of heart failure and mortality (10). Among anthracycline-induced arrhythmias, PVCs are one of the most common types, with a tendency for increased PVC burden following chemotherapy (11–14).

Traditional Chinese Medicine (TCM), as an important complementary therapy, is widely used in China and other Asian regions. TCM has shown promising benefits in mitigating cancer therapy-related cardiotoxicity and adverse gastrointestinal reactions, as well as improving quality of life (15). TCM syndrome (“Zheng” in Chinese), is an integral and essential part of TCM theory. A TCM syndrome is a predefined clinical phenotype or disharmony, which is a reproducible constellation of symptoms and signs derived from the four TCM examinations (inspection, auscultation/olfaction, inquiry, and pulse palpation). Clinical treatments of a patient rely on the successful differentiation of a specific TCM syndrome. A previous review has provided an in-depth description of the sequential relationship among symptom/sign, TCM syndrome, syndrome differentiation, and TCM treatment (16). *Qi* and *Yin* deficiency (QYD) syndrome is a specific TCM syndrome frequently observed in ACT. Patients with QYD syndrome typically present with symptoms such as palpitations, fatigue/low energy, shortness of breath, a reddened tongue, and a weak pulse. The fundamental TCM treatment principle for QYD syndrome is to “supplement *Qi* and nourish *Yin*,” with *Sheng Mai San*, a classical formula developed by the Jin Dynasty physician Zhang Yuansu, being its representative prescription (17). *Sheng Mai Yin* (SMY) is an oral liquid formulation derived from *Sheng Mai San*. As a licensed medication, it is indicated for the treatment of QYD syndrome-related clinical symptoms, including palpitations, shortness of breath, and spontaneous sweating. Clinical studies have demonstrated the protective effects of *Sheng Mai San* and its derivative oral and injectable formulations against anthracycline-induced cardiac dysfunction and arrhythmias, including PVCs (18, 19). Unfortunately, study design limitations, such as unreported randomization, allocation concealment, and blinding, have led to low-quality evidence in previous studies. Therefore, high-quality randomized controlled trials (RCTs) are necessary to rigorously evaluate the efficacy and safety of SMY.

To evaluate the efficacy and safety of SMY in the treatment of anthracycline-induced frequent PVCs, we plan to conduct a multicenter, randomized, double-blind, placebo-controlled clinical trial. The findings are expected to provide high-quality evidence to support SMY as an alternative or complementary therapy for anthracycline-induced frequent PVCs.

## 2 Methods and analysis

### 2.1 Study overview

**Figure 1:**
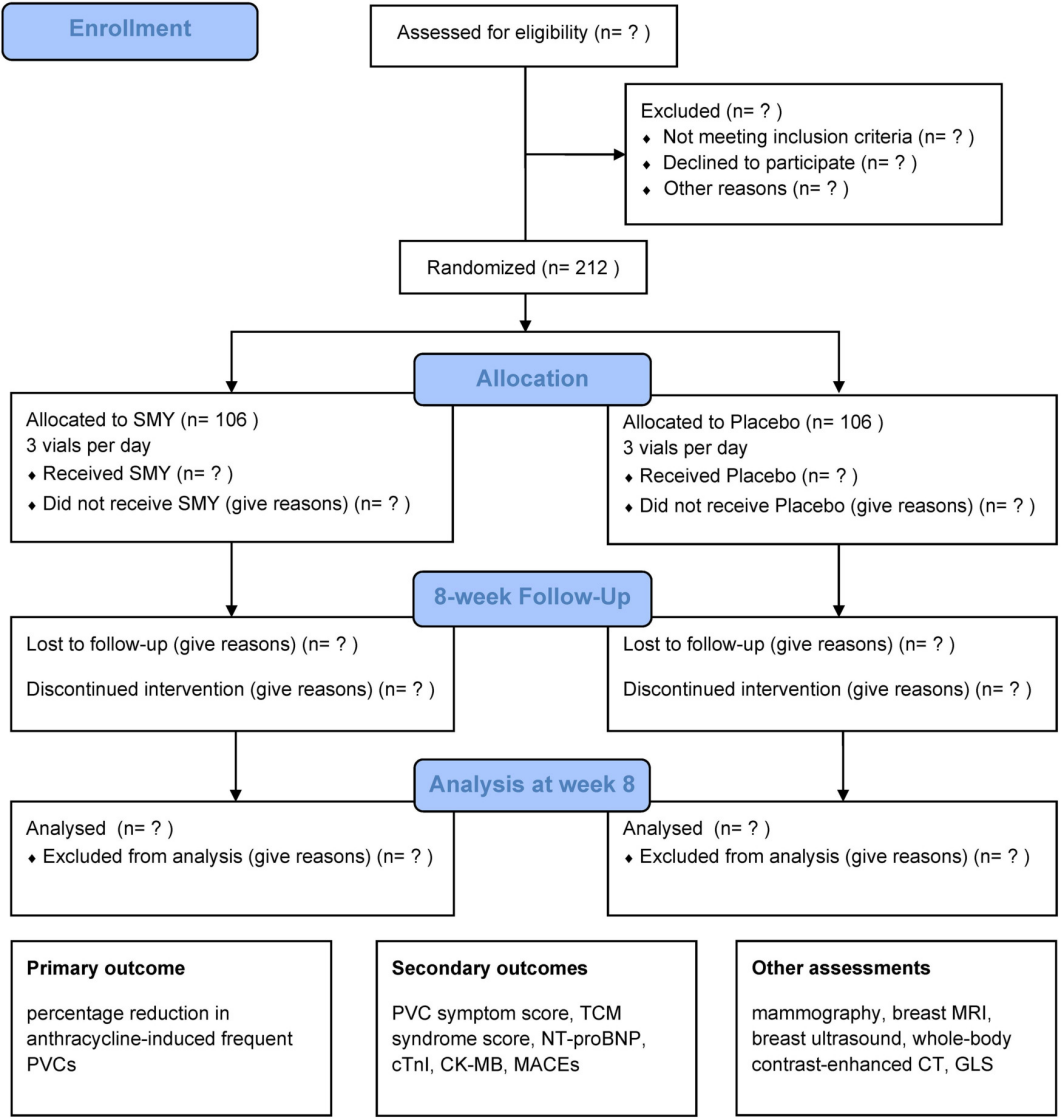
CONSORT-style flow diagram. SMY, *Sheng Mai Yin*; PVC, premature ventricular contraction; TCM, Traditional Chinese Medicine; NT-proBNP, N-terminal pro-brain natriuretic peptide; cTnI, cardiac troponin I; CK-MB, creatine kinase-MB; MACEs, major adverse cardiovascular events; MRI, magnetic resonance imaging; CT, computed tomography; GLS, global longitudinal strain.

This trial is a multicenter, randomized, double-blind, placebo-controlled clinical study designed to evaluate the efficacy and safety of SMY in treating frequent PVCs associated with QYD syndrome induced by anthracycline chemotherapy. We will screen participants among breast cancer and malignant lymphoma patients undergoing chemotherapy with anthracycline-based regimens. Eligible participants will be enrolled in the trial after providing written informed consent and will be randomly assigned in a 1:1 ratio to either the intervention group or the placebo control group. A total of 212 participants will be recruited for this trial, with both the treatment and follow-up periods lasting 8 weeks. The study design is illustrated in Figure 1.

In this trial, both SMY and its placebo are provided by Tong Ren Tang Technologies Co. Ltd., a Good Manufacturing Practice (GMP)-certified pharmaceutical company. Basic information on SMY is presented in Table 1. Given the botanical nature of SMY, the protocol has been developed in accordance with the Consensus-based reporting guidelines for Phytochemical Characterization of Medicinal Plant extracts (ConPhyMP) statement, to ensure transparency, reproducibility, and accurate interpretation of the study (20). Supplementary Appendix 1 provides comprehensive disclosure of SMY used in this trial, including its composition, sourcing, preparation, quality control, and other relevant aspects.

**Table 1:** Basic information on *Sheng Mai Yin*.

NMPA approval number	Batch number	Specification	Maximum daily dosage	Therapeutic indications	Components	Content per vial
Z11020363	24262304	10 mL per vial	30 mL	QYD syndrome with symptoms including palpitations, shortness of breath, and spontaneous sweating	Ginseng Radix et Rhizoma (Panax ginseng C. A. Mey., root and rhizome)	1,000 mg
					Ophiopogonis Radix [Ophiopogon japonicus (L. f) Ker-Gawl., root]	2,000 mg
					Schisandrae Chinensis Fructus [Schisandra chinensis (Turcz.) Baill., fruit]	1,000 mg

NMPA, national medical products administration; QYD syndrome, *Qi* and *Yin* deficiency syndrome.

The protocol is registered at itmctr.ccebtcm.org.cn (ITMCTR2024000858) on 29 September 2024 and is in full compliance with the principles of the Declaration of Helsinki and Good Clinical Practice (GCP) guidelines. The reporting of the protocol (Version 1.2—October 26, 2025) follows the Standard Protocol Items: Recommendations for Interventional Trials (SPIRIT) guidelines (20). The SPIRIT checklist is available in Supplementary Table S1. Three hospitals have agreed to participate in the trial and have each received ethical approval from their respective ethics committees: Dongzhimen Hospital, Beijing University of Chinese Medicine (ID: 2024DZMEC-438-02); Peking University Third Hospital (ID: S20241000); and the Institute of Hematology & Blood Disease Hospital, Chinese Academy of Medical Sciences, Peking Union Medical College (ID: NKRDP2024017-EC-1).

### 2.2 Diagnostic criteria for breast cancer and malignant lymphoma

The diagnostic criteria for breast cancer and malignant lymphoma are based on the “Guidelines for Breast Cancer Diagnosis and Treatment by the China Anti-Cancer Association (2024 edition)” (21) and the “Chinese Society of Clinical Oncology (CSCO) Diagnosis and Treatment Guidelines for Malignant Lymphoma (2021)” (22). Diagnosis must be pathologically confirmed, and original pathology reports will be retained.

### 2.3 Definition of frequent PVCs

The diagnostic criteria for PVCs are based on the “Chinese Expert Consensus on Ventricular Arrhythmia (2016 Consensus Upgrade Version)” (23). There is currently no unified standard for the definition of frequent PVCs. Based on the Chinese expert consensus (23), we define frequent PVCs as follows: more than 500 PVCs detected on 24 h Holter monitor or the presence of paired or consecutive PVCs.

### 2.4 Diagnostic criteria for QYD syndrome

The diagnostic criteria for QYD syndrome are based on the “Guiding Principles for Clinical Study of New Chinese Medicines” (24) and the China National Standard GB/T 16751.2-2021, titled “Clinical Terminology of Traditional Chinese Medical Diagnosis and Treatment—Part 2: Syndromes/Patterns”. The main symptoms of QYD syndrome include: dizziness, palpitations, insufficiency of spirit, and shortness of breath. The secondary symptoms are: excessive dreaming, red cheeks, dry mouth, feverish sensations in the palms, soles, and chest, spontaneous sweating, and night sweats. Tongue and pulse conditions include: red tongue, thin coating, weak and/or rapid pulse. A diagnosis of QYD syndrome can be made if the participant meets at least one main symptom and two secondary symptoms, or two main symptoms and one secondary symptom, along with the characteristic tongue and pulse conditions. The definitions or descriptions of the symptoms mentioned above are provided in Table 2. TCM syndromes of each participant will be independently evaluated by two experienced TCM practitioners. In cases of diagnostic disagreement, the final diagnosis will be made by the chief physician based on clinical data. The initial diagnoses made by the two practitioners will be recorded, and after all participants have been enrolled, Kappa statistics will be used to test the consistency of the diagnoses. A *κ* value greater than 0.6 will be considered acceptable.

**Table 2:** TCM syndrome score—Qi and Yin deficiency syndrome.

Symptom	Description/Definition	None	Mild	Moderate	Severe
Dizziness	A common symptom that includes vertigo, nonspecific dizziness, disequilibrium, and/or presyncope.	0	1	2	3
Palpitations	Unpleasant, involuntary sensations of rapid, irregular and/or forceful beating of the heart.	0	1	2	3
Insufficiency of spirit	Dull eyes with sluggish eyeballs, poor spirit, lassitude, slow response, a complexion lacking luster, flabby muscle and slow body movements.	0	1	2	3
Shortness of breath	A subjective experience of rapid breathing and shortness of breath that resembles panting without elevated shoulders or phlegm sounds in the throat.	0	1	2	3
Excessive dreaming	Interrupted sleep by strange dreams or frightening nightmares. Individuals often have a clear memory of the dreams.	0	1	2	3
Red cheeks	Reddening of the cheeks only.	0	1	2	3
Dry mouth	A subjective feeling of insufficient saliva in the mouth.	0	1	2	3
Feverish sensations in palms, soles and chest	A subjective feeling of restlessness and heat sensation in the chest, along with feverish sensations in the palms and soles.	0	1	2	3
Spontaneous sweating	Characterized by persistent, spontaneous day sweating that is not attributed to physical exertion, hot weather, wearing too many clothes or taking diaphoretics.	0	1	2	3
Night sweats	Characterized by episodes of sweating during sleep that stop after waking up and are not related to external environmental factors.	0	1	2	3
Red tongue	A tongue redder than normal.	0	1	2	3
Thin coating	A tongue coating (the layer of moss-like material covering the tongue) through which the underlying tongue surface is faintly visible.	0	1	2	3
Weak pulse	A pulse that feels thready, soft, deep and weak.	0	1	2	3
Rapid pulse	A pulse with more than 5 beats in one breath (>75 bpm).	0	1	2	3

TCM, Traditional Chinese Medicine.

### 2.5 Inclusion criteria

1. Age 18–75 years.
2. Patients with breast cancer or malignant lymphoma who develop frequent PVCs after receiving anthracycline therapy: those with more than 500 PVCs per 24 h or those with paired or consecutive PVCs.
3. TCM diagnosis of QYD syndrome.
4. Voluntary signing of informed consent.

### 2.6 Exclusion criteria

1. Severe coronary artery disease or heart valve disease that is difficult to control with medication, or associated with hemodynamic instability.
2. Pacemaker or implantable cardioverter defibrillator (ICD) implantation.
3. Severe complications related to the primary cancer, potentially life-threatening.
4. History of bleeding within the past month, including cerebrovascular, gastrointestinal, respiratory, urinary tract, or other organ bleeding.
5. Severe psychiatric disorders.
6. Allergy to any components of the study drug.
7. Unexplained persistent elevation of transaminases (greater than three times the upper limit of normal).
8. Severe renal dysfunction: estimated glomerular filtration rate (eGFR) < 45 mL/(min·1.73 m^2^).
9. Severe infection.
10. Pregnancy or breastfeeding.
11. Concurrent oral cyclosporine use.
12. Life expectancy of less than 2 years.
13. Participation in other clinical trials within the past month.

### 2.7 Termination criteria

The treatment should be considered for discontinuation under the following conditions:

1. The participant has the right to discontinue treatment at any time and for any reason.
2. An allergic reaction clearly related to the study drug occurs.
3. Adverse symptoms, signs, or abnormal test results clearly related to the study drug occur.
4. The investigator determines that discontinuation of the study is necessary.
5. Pregnancy occurs during the study in a female participant.

Throughout the trial, efforts should be made to ensure that participants receive the standard dose of the study drug for the long term. Patients who discontinue the study drug should resume treatment as soon as possible after ruling out the aforementioned conditions and continue follow-up as scheduled.

### 2.8 Withdrawal criteria

All eligible participants have the right to withdraw from the study at any time and for any reason. However, unnecessary withdrawals should be minimized. When a participant decides to withdraw, we will contact the patient or their relatives via telephone or in-person visits to confirm the reason for withdrawal whenever possible. Upon withdrawal, the remaining study drug will be collected, and a final assessment will be conducted. Efforts will be made to complete the case report form (CRF), document the reason for withdrawal, and follow up on endpoint events in withdrawn patients. If the withdrawal is due to an adverse event, it must be recorded in the CRF.

### 2.9 Screening and recruitment of participants

We will conduct regular evaluations of breast cancer and malignant lymphoma patients undergoing anthracycline-containing chemotherapy regimens in the outpatient and inpatient departments of three hospitals. We will implement the following screening workflow to maximize identification of eligible participants. (1) Pre-chemotherapy: 24 h Holter will be performed before the first chemotherapy cycle, together with medical history review, to rule out pre-existing frequent PVCs. (2) During chemotherapy: 12-lead ECG will be obtained at each cycle. Additionally, 24 h Holter will be repeated every four cycles, and whenever clinically indicated (triggered by abnormal ECG or symptoms). (3) Post-chemotherapy follow-up: 12-lead ECG will be performed at each scheduled follow-up visit, and 24 h Holter will be repeated if clinically indicated (abnormal ECG or symptoms).

Based on the inclusion and exclusion criteria, eligible participants will be recruited. Each patient will receive either a printed or electronic version of the recruitment advertisement. The advertisement will provide a brief description of the study objectives, procedures, potential benefits, and risks. Each eligible participant will sign a consent form provided by study personnel prior to enrollment. Participants will not be involved in the design, conduct, and reporting of the trial.

### 2.10 Sample size

A previous RCT demonstrated that after 4 weeks of treatment, the number of PVCs within 24 h decreased from 15,138 ± 7,597 to 5,686 ± 5,940 in the intervention group. While in the placebo group, the number decreased from 14,529 ± 5,929 to 10,592 ± 8,009 (25). The reduction in PVC counts for both groups was converted into reduction rates, which were 62.44% ± 39.24% in the intervention group and 27.10% ± 55.12% in the placebo group. The standard deviation (SD) of the reduction rate was estimated by dividing the reported post-treatment SD of PVC counts by the baseline mean number of PVCs.

Based on these findings, we assumed that the mean percentage reduction in 24 h PVC counts would be 60% in the intervention group and 35% in the placebo group, with SDs of 39% and 55%, respectively. To detect a between-group difference of 25% in the percentage reduction rate with 95% power at a two-sided significance level of 5%, a total of 95 participants per group would be required. Allowing for a 10% loss to follow-up, the final required sample size was set at 212 participants, with 106 participants allocated to each group.

Each participating center has a high volume of patients. Adequate study personnel will be assigned to screen, monitor, and recruit potential participants to ensure that the target sample size is achieved.

### 2.11 Randomization and blinding

Participants will be randomly assigned to either the intervention group (SMY group) or the placebo control group in a 1:1 ratio using a centralized web-based randomization system. Randomization will be stratified by study center, and the sequence will be computer-generated with permuted blocks of variable sizes within each center to ensure balanced allocation while minimizing predictability. The allocation sequence will remain concealed from participants and study personnel, including physicians, investigators, data collectors, and statisticians, to prevent selection bias.

This trial will be conducted in a double-blind manner, ensuring that both participants and study personnel remain unaware of treatment assignments throughout the trial. To ensure effective blinding, SMY and its placebo will be designed to be indistinguishable in appearance, smell, taste, and packaging.

### 2.12 Unblinding

An independent Data Monitoring Committee (DMC) will conduct periodic safety reviews and will have access to unblinded safety data to ensure patient safety and trial integrity. The study investigators and other study personnel will remain blinded unless emergency unblinding is deemed necessary for patient safety. The decision to unblind a participant will be made by the principal investigator or the DMC, based on predefined criteria.

Unblinding will be conducted through the centralized randomization system, which maintains a secure and auditable record of all unblinding requests. In the event of unblinding, the reason, date, and responsible personnel will be documented, and its potential impact on study outcomes will be assessed.

Routine unblinding will not be performed before database lock and statistical analysis. The randomization codes will remain concealed until the completion of data analysis, except for cases of authorized emergency unblinding.

### 2.13 Interventions

Participants will receive treatment for 8 weeks. During the treatment period, participants are not allowed to take any other TCM containing components similar to those in SMY.

#### 2.13.1 Intervention group (SMY group)

Participants randomized to the intervention group will receive standardized Western medicine treatment in combination with SMY. SMY will be administered orally at a dose of 1 vial, three times daily (1 vial, TID, PO).

#### 2.13.2 Placebo control group

Participants randomized to the placebo control group will receive standardized Western medicine treatment in combination with a placebo matched to SMY. The placebo will be identical in appearance, smell, taste, and packaging to SMY, ensuring effective blinding. It will be administered orally at the same dose and frequency as SMY (1 vial, TID, PO).

#### 2.13.3 Standardized western medicine treatment

All participants will receive standardized Western medicine treatment individualized to clinical conditions, including:

1. Physician-determined chemotherapy regimens incorporating anthracyclines (21, 22).
2. Primary prevention of ACT, recommended by clinical guidelines (26), may include dexrazoxane, angiotensin-converting enzyme inhibitors (ACEIs) or angiotensin II receptor blockers (ARBs), β-blockers, and/or statins, tailored to individual patient risk profiles.
3. Guideline-directed therapy for PVCs (23). For most symptomatic patients, β-blockers are the first-line treatment. Other standard options, including antiarrhythmic drugs (AADs) and catheter ablation in selected cases, are permitted as clinically indicated.
4. Medications for hypertension, diabetes, dyslipidemia, and other comorbidities, as clinically indicated.

#### 2.13.4 Drug dispensation and retrieval

SMY and its placebo will be dispensed by trained study personnel at each follow-up visit. Participants will be instructed to return all used and unused vials at each follow-up visit, and medication compliance will be assessed through vial counts and participant self-reports. The actual amount of medication taken within the range of 80% to 100% of the prescribed dosage is considered to meet the protocol requirements for medication compliance.

### 2.14 Outcome measurements

#### 2.14.1 Primary outcome

Percentage reduction in anthracycline-induced frequent PVCs, which is calculated by subtracting the post-treatment 24 h PVC count at 8 weeks from the baseline 24 h PVC count, then dividing the result by the baseline 24 h PVC count, and multiplying by 100%.

#### 2.14.2 Secondary outcomes

1. PVC symptom score at 8 weeks**:** Currently, there is no standardized scale for PVC symptom assessment. Based on expert opinions, we have developed a scale to preliminarily evaluate the impact of anthracycline-induced frequent PVCs on participants and to assess changes in symptom scores before and after SMY treatment. The scoring will be based on patient self-reports and will include the following symptoms: palpitations, dizziness, chest pain, insomnia, and fatigue. Each symptom will be classified into four levels according to its frequency and severity: none, mild, moderate, and severe, corresponding to symptom scores of 0, 1, 2, and 3, respectively.
2. TCM syndrome score at 8 weeks: Based on the diagnostic criteria for QYD syndrome and expert opinions, we have developed the TCM syndrome score, which consider symptom severity as well as tongue and pulse conditions. The detailed scoring criteria are provided in Table 2. Experienced TCM practitioners will assess patients' clinical manifestations and incorporate self-reported symptoms to determine the scores.
3. Cardiac dysfunction biomarkers at 8 weeks: Levels of N-terminal pro-brain natriuretic peptide (NT-proBNP), cardiac troponin I (cTnI), and creatine kinase-MB (CK-MB).
4. Major adverse cardiovascular events (MACEs): Including cardiac death, myocardial infarction, stroke, severe heart failure, and unstable angina requiring hospitalization or revascularization.

#### 2.14.3 Safety outcomes and adverse events

1. Vital Signs: Temperature, blood pressure, respiration, and heart rate.
2. Blood routine test, urine routine test, and stool routine test.
3. Electrocardiogram (ECG), liver function, renal function, and coagulation function, including alanine aminotransferase (ALT), aspartate aminotransferase (AST), total bilirubin (TBIL), direct bilirubin (DBIL), indirect bilirubin (IBIL), blood urea nitrogen (BUN), creatinine (Cr), prothrombin time (PT) and activated partial thromboplastin time (APTT).
4. Adverse events (AEs): Any unfavorable medical event occurring from the time the participant signs the informed consent form until the final follow-up visit, regardless of its causal relationship with the study drug, will be classified as an AE and must be recorded in the CRF. All AEs should be continuously followed up until they disappear or stabilize.

#### 2.14.4 Baseline assessments

Baseline data of participants will be recorded before the initiation of treatment:

1. Demographic information: Gender, age, medical record number, height, weight, smoking history, alcohol consumption history, and study center.
2. Breast cancer or malignant lymphoma information: Pathological classification, breast cancer receptor status, staging, thoracic radiotherapy (laterality and total dose), chemotherapy regimen, cumulative dose of anthracyclines, and primary prevention medications for ACT.
3. Comorbidities and relevant medications: Including hypertension, diabetes, dyslipidemia, coronary artery disease, and cardiomyopathy.
4. TCM diagnostic information: Including diagnostic symptoms, tongue characteristics, and pulse conditions.

#### 2.14.5 Other assessments

1. Tumor progression assessment: Including mammography, breast magnetic resonance imaging (MRI), breast ultrasound, and whole-body contrast-enhanced computed tomography (CT).
2. High-sensitivity predictive biomarker for ACT: Global longitudinal strain (GLS) is one of the most promising indicators of cardiac function assessment after left ventricular ejection fraction (LVEF). Its relative change is an ideal tool for identifying asymptomatic cancer therapy-related cardiac dysfunction (CTRCD) and has greater sensitivity than LVEF (27, 28). Therefore, GLS is selected as the high-sensitivity predictive biomarker for ACT.

### 2.15 Study visit

The follow-up period for this study will last for 8 weeks, with follow-up assessments conducted at baseline, week 3, and week 8. At baseline and week 8, questionnaires and clinical examinations will be conducted. At week 3 and week 8, AEs, MACE, drug dispensation, and drug retrieval will also be conducted. The detailed schedule of visits and data collection is listed in Table 3.

**Table 3:** Timeline of visits and data collection.

Items	Visit		
	Baseline (−3 d to 0 d)	Week 3 (±3 d)	Week 8 (±3 d)
Informed consent signing	√		
Inclusion/exclusion criteria	√		
Randomization	√		
Demographic information	√		
Breast cancer or lymphoma treatment information	√		
Comorbidities and concomitant medication	√		
TCM diagnosis	√		
24 h Holter monitor	√		√
PVC symptom score	√		√
TCM syndrome score	√		√
NT-proBNP, cTnI, and CK-MB	√		√
MACEs	√	√	√
Mammography	√		√
Breast MRI	√		√
Breast ultrasound	√		√
Whole-body contrast-enhanced CT	√		√
GLS	√		√
Vital signs	√		√
Routine blood, urine, and stool tests	√		√
ECG	√		√
Liver and renal function	√		√
Coagulation function	√		√
Adverse events		√	√
Drug dispensation	√	√	
Drug retrieval		√	√

PVC, premature ventricular contraction; TCM, Traditional Chinese Medicine; NT-proBNP, N-terminal pro-brain natriuretic peptide; cTnI, cardiac troponin I; CK-MB, creatine kinase-MB; MACEs, major adverse cardiovascular events; GLS, global longitudinal strain; ECG, electrocardiogram.

### 2.16 Retention

Successful completion of evaluation requirements for the study is defined as completing assessments of at least two time periods (baseline and week 8 follow-up). However, participants may withdraw from the study at any time for any reason. Participant retention, termination, withdrawal, and loss to follow-up will be documented and discussed.

### 2.17 Statistical analysis plan

Primary efficacy outcome will be analyzed using a linear model (LM). The primary LM model will include the treatment group, study center, and baseline 24 h PVC count. A fully adjusted LM model analysis will also be performed by incorporating pre-specified baseline covariates into the LM model. These covariates include sex, age, BMI, cancer type, thoracic radiotherapy, cumulative dose of anthracyclines, and the presence of any cardiovascular risk factors (hypertension, diabetes, dyslipidemia, coronary artery disease, cardiomyopathy, smoking, and alcohol consumption). The unadjusted and adjusted mean difference between intervention and control in the primary outcome, together with its 95% confidence intervals (CIs) will be derived from the LM models. In addition, subgroup analysis of the primary endpoint will be performed on the pre-specified covariates including baseline 24 h PVC burden which will be examined in quartiles (*Q*1–*Q*4) as a pre-specified subgroup. Treatment heterogeneity will be assessed by fitting a treatment-by-subgroup interaction using a Wald test. Normality assumption for the residuals will be assessed using *Q–Q* plot. The win ratio method will be employed if normality assumption is violated. Missing data will be imputed using the multiple imputation method.

Analysis of secondary continuous outcomes and GLS will be performed in a similar way to the primary outcome analysis. Analysis of secondary binary outcomes will be performed using generalized linear model (GLM) models with binomial distribution and log function. The risk ratio with 95% CIs between intervention and control will be derived from the GLM model.

Exploratory analyses: The associations between (i) baseline TCM syndrome score and baseline 24 h PVC count, and (ii) change in TCM syndrome score and the percentage reduction in PVCs will be assessed. For each association, Spearman's rank correlation will be computed. For (ii), a LM will be fitted in a similar way to the models for continuous outcomes.

Analysis of safety outcomes and AEs: AEs will be summarized as the number of cases and incidence rates, with comparisons made between groups. Abnormalities in vital signs, liver function, renal function, and coagulation function after treatment will be summarized using contingency table, and differences between groups will be analyzed.

All statistical analyses will be performed using SPSS Version 26.0 (IBM Corp, Armonk, NY, USA) and R version 4.1.1. The trial results will be reported following the Consolidated Standards of Reporting Trials (CONSORT) guidelines for reporting randomized trials (29).

Primary data analyses will be based on the intention-to-treat principle. The per-protocol analyses will also be performed as supplemental analysis. Descriptive statistics will be produced for outcome variables and for baseline characteristics of patients by treatment arm. Continuous variables will be summarized using number of observations, mean (SD) or median (IQR) depending on their distributions; categorical variables will be summarized by the number and percentage.

No interim analysis of outcomes is planned. No multiple adjustment will be made.

### 2.18 Quality control

To ensure the quality of this trial, a multicenter trial coordination committee and an overall principal investigator will be established. The coordination committee, consisting of principal investigators and key researchers from each center, will be responsible for addressing issues arising during the trial. All researchers and staff involved in this study will undergo training based on standard operating procedures (SOPs) to ensure a thorough understanding of the trial's objectives and procedures. To reduce inter-center variability in the diagnosis of TCM syndrome and scoring, each site will employ a fixed roster of TCM practitioners. Prior to randomization, all TCM practitioners will complete centralized training. A unified inquiry template and scripted prompts will be used for both diagnosis and scoring to standardize information elicitation across centers. When a replacement is unavoidable, the incoming TCM practitioner will complete the same training before assessing participants. The DMC will appoint inspectors to conduct regular on-site monitoring (no less than once per month) to safeguard participant rights, ensure compliance with the trial protocol, and maintain data accuracy.

The CRFs will be independently entered into the online database by two data collectors, and the data will be reviewed and verified by the DMC before being locked. All information about participants will be anonymized and will be kept confidential. The database will be password-protected and managed by the DMC. Complete data will only be accessible during the data analysis phase by study investigators and statisticians.

SMY (Tong Ren Tang Technologies Co., Ltd.) will be supplied as a single lot (Batch No. 24262304) across all sites. Schisandrol A content will be quantified by High-performance liquid chromatography (HPLC) to meet the pharmacopeial threshold (≥0.25 mg/10 mL) (30). Full quality control strategies are detailed in Supplementary Appendix 1.

Adjustments to the protocol are possible in terms of technical details derived by the pilot testing.

## 3 Discussion

ACT could not only cause cardiac damage, but also limit the implementation of chemotherapy regimens. Therefore, mitigating cardiotoxicity is crucial for improving prognosis. As an important alternative and complementary therapy, TCM holds promise in the prevention and treatment of ACT. However, high-quality clinical evidence is still needed.

Calcium plays an important role in the pathogenesis of various arrhythmias, and anthracycline-induced arrhythmias are no exception. The key mechanisms by which anthracyclines induce arrhythmias include the regulation of Ca^2+^/calmodulin-dependent protein kinase II (CaMKII), ryanodine receptor 2 (RyR2), and L-type calcium channels, leading to calcium overload and ultimately resulting in arrhythmias (31–33). As one of the key components of SMY, Ginseng contains multiple active compounds that exert anti-arrhythmic effects via calcium channel regulation. For example, Ginsenoside Rg2 can downregulate CaMKII phosphorylation, reduce L-type calcium channel (LTCC)-mediated Ca^2+^ influx, and decrease both the incidence and duration of malignant arrhythmias (34). Additionally, Ginsenoside Rg3 can inhibit calcium overload by enhancing the activity of sarcoplasmic/endoplasmic reticulum Ca^2+^ ATPase 2a (SERCA2a) (35). Moreover, Ophiopogonin D (OP-D) and Schisandrin B (Sch B) have been shown to alleviate doxorubicin-induced cardiotoxicity through their antioxidant and anti-inflammatory effects (36, 37). Anthracyclines can induce the production of reactive oxygen species (ROS), which excessively activate CaMKII, leading to calcium overload (38, 39). Therefore, OP-D and Sch B may indirectly improve arrhythmia by mitigating ROS-induced CaMKII overactivation. These mechanisms support the potential value of SMY in the treatment of anthracycline-induced frequent PVCs. However, its efficacy and safety still require further validation through large-scale, multicenter RCTs. Previous studies have explored the mechanisms by which SMY mitigates anthracycline-induced myocardial injury and cardiac dysfunction (40, 41). However, further mechanistic investigations are warranted to elucidate how SMY specifically modulates anthracycline-related arrhythmia.

Previous clinical studies have provided limited support for the efficacy of SMY in mitigating ACT. A meta-analysis that included 19 RCTs (*n* = 2,331) suggested that SMY improved anthracycline-induced arrhythmias, left-ventricular function, and myocardial injury (18). A subsequent meta-analysis of 16 RCTs (*n* = 2,140) indicated that the injection formulation of SMY reduced anthracycline-induced arrhythmias and myocardial injury but did not improve left-ventricular ejection fraction (LVEF) (19). Because most clinical trials did not report safety outcomes, both meta-analyses failed to assess the safety profile of SMY. Meanwhile, most clinical trials are of limited methodological quality, lack systematic safety evaluation, and are published almost exclusively in Chinese. These limitations hinder broad international recognition and the development of high-quality evidence. In addition, definitions of anthracycline-induced arrhythmia varied considerably; many clinical trials included nonspecific ECG abnormalities (e.g., ST-T segment changes, low QRS voltage) and occasional atrial or ventricular premature contractions. To date, no clinical trial has specifically evaluated the efficacy and safety of SMY for anthracycline-induced frequent PVCs.

This trial aims to address the limitations of previous studies and clarify the efficacy and safety of SMY. The primary outcome is the percentage reduction in PVCs at 8 weeks. The TCM syndrome score is a pre-specified secondary, patient-centered outcome, which comprises many non-specific symptoms. Its association with PVC burden will be evaluated exploratorily and will not affect measurement and inference on the primary outcome. The exploratory analyses are designed to assess whether the TCM syndrome score tends to improve in parallel with reductions in PVC burden, rather than to investigate a causal relationship between the two. Additionally, we will investigate the potential effects of SMY on cardiac dysfunction, with relevant indicators including NT-proBNP, cTnI, CK-MB, and GLS (27). Based on previous studies and clinical experience (19), an 8-week treatment course will be set to evaluate the efficacy of SMY. If this trial demonstrates the effectiveness of SMY, further studies will be considered, including an extended follow-up period to assess its long-term efficacy, as well as an evaluation of its effects in patients without QYD syndrome.

This trial has several limitations. Notably, although there is no universally accepted cut-off for defining “frequent PVCs,” contemporary Western guidelines commonly recommend ≥10,000 PVCs per day or a PVC burden >10% as the threshold (42, 43). In contrast, in accordance with the Chinese Expert Consensus, we adopted a lower threshold (>500 PVCs per day) to define frequent PVCs (23). This choice aims to better reflect real-world cardio-oncology practice in China and avoid unduly restricting generalizability to Chinese populations. To aid interpretability and comparability, we have pre-specified subgroup analyses by baseline PVC-burden quartiles and will evaluate the treatment-by-quartile interaction, allowing us to assess treatment effects across the full spectrum of baseline PVC burden. Second, this trial will be conducted only in Beijing and Tianjin, which limits the generalizability of SMY's efficacy to other populations and regions. Third, among the secondary outcomes, we will use unvalidated PVC symptom score and TCM syndrome score. However, these measures can still provide insight into changes in participants before and after treatment. Finally, this trial will include only patients with QYD syndrome, which may introduce selection bias.
