# Supplementary material for: Investigation of Sheng Mai Yin in the treatment of anthracycline-induced frequent premature ventricular contractions in cancer patients: study protocol for a multicenter, randomized, double-blind, placebo-controlled clinical trial
Source: Front Cardiovasc Med. 2025 Nov 24;12:1670053. doi: 10.3389/fcvm.2025.1670053 (PMC12682750; doi:10.3389/fcvm.2025.1670053)
Supplement: Supplementary file 1 [file Datasheet1.pdf]

Supplementary Material

Contents

Supplementary Table S1 The SPIRIT 2025 checklist

Appendix 1 Detailed Information on *Sheng Mai Yin*

References

Supplementary Table S1 The SPIRIT 2025 checklist

Section / Topic	No	SPIRIT 2025 checklist item description	Reported on page no. & section headings
**Administrative information**			
Title and structured summary	1a	Title stating the trial design, population, and interventions, with identification as a protocol	Title page
	1b	Structured summary of trial design and methods, including items from the World Health Organization Trial Registration Data Set	Abstract
Protocol version	2	Version date and identifier	Study overview
Roles and responsibilities	3a	Names, affiliations, and roles of protocol contributors	Title page; Author Contributions
	3b	Name and contact information for the trial sponsor	Funding
	3c	Role of trial sponsor and funders in design, conduct, analysis, and reporting of trial; including any authority over these activities	Funding; Conflict of interest
	3d	Composition, roles, and responsibilities of the coordinating site, steering committee, endpoint adjudication committee, data management team, and other individuals or groups overseeing the trial, if applicable	Study overview; Unblinding; Quality control
**Open science**			
Trial registration	4	Name of trial registry, identifying number (with URL), and date of registration. If not yet registered, name of intended registry	Abstract
Protocol and statistical analysis plan	5	Where the trial protocol and statistical analysis plan can be accessed	Abstract; Statistical analysis plan
Data sharing	6	Where and how the individual de-identified participant data (including data dictionary), statistical code, and any other materials will be accessible	Ethics and dissemination
Funding and conflicts of interest	7a	Sources of funding and other support (e.g., supply of drugs)	Funding; Conflict of interest
	7b	Financial and other conflicts of interest for principal investigators and steering committee members	Conflict of interest
Dissemination policy	8	Plans to communicate trial results to participants, healthcare professionals, the public, and other relevant groups (e.g., reporting in trial registry, plain language summary, publication)	Ethics and dissemination
**Introduction**			
Background and rationale	9a	Scientific background and rationale, including summary of relevant studies (published and unpublished) examining benefits and harms for each intervention	Introduction
	9b	Explanation for choice of comparator	Introduction
Objectives	10	Specific objectives related to benefits and harms	Introduction
**Methods: Patient and public involvement, trial design**			
Patient and public involvement	11	Details of, or plans for, patient or public involvement in the design, conduct, and reporting of the trial	Screening and recruitment of participants
Trial design	12	Description of trial design including type of trial (e.g., parallel group, crossover), allocation ratio, and framework (e.g., superiority, equivalence, non-inferiority, exploratory)	Study overview
**Methods: Participants, interventions, and outcomes**			

Trial setting	13	Settings (e.g., community, hospital) and locations (e.g., countries, sites) where the trial will be conducted	Study overview
Eligibility criteria	14a	Eligibility criteria for participants	Inclusion criteria; Exclusion criteria
	14b	If applicable, eligibility criteria for sites and for individuals who will deliver the interventions (e.g., surgeons, physiotherapists)	Quality control
Intervention and comparator	15a	Intervention and comparator with sufficient details to allow replication including how, when, and by whom they will be administered. If relevant, where additional materials describing the intervention and comparator (e.g., intervention manual) can be accessed	Interventions; Appendix 1
	15b	Criteria for discontinuing or modifying allocated intervention/comparator for a trial participant (e.g., drug dose change in response to harms, participant request, or improving/worsening disease)	Termination criteria; Withdrawal criteria
	15c	Strategies to improve adherence to intervention/comparator protocols, if applicable, and any procedures for monitoring adherence (e.g., drug tablet return, sessions attended)	Termination criteria; Withdrawal criteria; Drug dispensation and retrieval
	15d	Concomitant care that is permitted or prohibited during the trial	Interventions
Outcomes	16	Primary and secondary outcomes, including the specific measurement variable (e.g., systolic blood pressure), analysis metric (e.g., change from baseline, final value, time to event), method of aggregation (e.g., median, proportion), and time point for each outcome	Primary outcome; Secondary outcomes
Harms	17	How harms are defined and will be assessed (e.g., systematically, non-systematically)	Safety outcomes and adverse events
Participant timeline	18	Time schedule of enrollment, interventions (including any run-ins and washouts), assessments, and visits for participants. A schematic diagram is highly recommended (see Figure)	Figure 1
Sample size	19	How sample size was determined, including all assumptions supporting the sample size calculation	Sample size
Recruitment	20	Strategies for achieving adequate participant enrollment to reach target sample size	Screening and recruitment of participants
**Methods: Assignment of interventions**			
Randomization:			
Sequence generation	21a	Who will generate the random allocation sequence and the method used	Randomization and blinding
	21b	Type of randomization (simple or restricted) and details of any factors for stratification. To reduce predictability of a random sequence, other details of any planned restriction (e.g., blocking) should be provided in a separate document that is unavailable to those who enroll participants or assign interventions	Randomization and blinding
Allocation concealment mechanism	22	Mechanism used to implement the random allocation sequence (e.g., central computer/telephone; sequentially numbered, opaque, sealed containers), describing any steps to conceal the sequence until interventions are assigned	Randomization and blinding
Implementation	23	Whether the personnel who will enroll and those who will assign participants to the interventions will have access to the random allocation sequence	Randomization and blinding; Unblinding
Blinding	24a	Who will be blinded after assignment to interventions (e.g., participants, care providers, outcome assessors, data analysts)	Randomization and blinding

	24b	If blinded, how blinding will be achieved and description of the similarity of interventions	Randomization and blinding
	24c	If blinded, circumstances under which unblinding is permissible, and procedure for revealing a participant's allocated intervention during the trial	Unblinding
**Methods: Data collection, management, and analysis**			
Data collection methods	25a	Plans for assessment and collection of trial data, including any related processes to promote data quality (e.g., duplicate measurements, training of assessors) and a description of trial instruments (e.g., questionnaires, laboratory tests) along with their reliability and validity, if known. Reference to where data collection forms can be accessed, if not in the protocol	Outcome measurements
	25b	Plans to promote participant retention and complete follow-up, including list of any outcome data to be collected for participants who discontinue or deviate from intervention protocols	Withdrawal criteria; Retention
Data management	26	Plans for data entry, coding, security, and storage, including any related processes to promote data quality (e.g., double data entry; range checks for data values). Reference to where details of data management procedures can be accessed, if not in the protocol	Quality control
Statistical methods	27a	Statistical methods used to compare groups for primary and secondary outcomes, including harms	Statistical analysis plan
	27b	Definition of who will be included in each analysis (e.g., all randomized participants), and in which group	Statistical analysis plan
	27c	How missing data will be handled in the analysis	Statistical analysis plan
	27d	Methods for any additional analyses (e.g., subgroup and sensitivity analyses)	Statistical analysis plan
**Methods: Monitoring**			
Data monitoring committee	28a	Composition of data monitoring committee (DMC); summary of its role and reporting structure; statement of whether it is independent from the sponsor and funder; conflicts of interest and reference to where further details about its charter can be found, if not in the protocol. Alternatively, an explanation of why a DMC is not needed	Unblinding; Quality control
	28b	Explanation of any interim analyses and stopping guidelines, including who will have access to these interim results and make the final decision to terminate the trial	Statistical analysis plan
Trial monitoring	29	Frequency and procedures for monitoring trial conduct. If there is no monitoring, give explanation	Quality control
**Ethics**			
Research ethics approval	30	Plans for seeking research ethics committee/institutional review board approval	Study overview; Ethics and dissemination
Protocol amendments	31	Plans for communicating important protocol modifications to relevant parties	NA
Consent or assent	32a	Who will obtain informed consent or assent from potential trial participants or authorized proxies, and how	Screening and recruitment of participants
	32b	Additional consent provisions for collection and use of participant data and biological specimens in ancillary studies, if applicable	NA
Confidentiality	33	How personal information about potential and enrolled participants will be collected, shared, and maintained in order to protect confidentiality before, during, and after the trial	Quality control
Ancillary and post-trial care	34	Provisions, if any, for ancillary and post-trial care, and for compensation to those who suffer harm from trial participation	Ethics and dissemination

© 2025 Chan A-W et al. This is an Open Access article distributed under the terms of the Creative Commons Attribution License (https://creativecommons.org/licenses/by/4.0/), which permits unrestricted use, distribution, and reproduction in any medium, provided the original work is properly cited.

Appendix 1 Detailed Information on *Sheng Mai Yin*

This appendix provides comprehensive information on *Sheng Mai Yin* (SMY). The content is structured in accordance with the Consensus-based reporting guidelines for Phytochemical Characterisation of Medicinal Plant extracts (ConPhyMP) statement, to ensure the transparency, reproducibility, and accurate interpretation of our study (1).

1. Herbal components

A summary of the herbal components of SMY is provided in **Table A1**. Detailed chemical constituents of these herbal components have been well elaborated elsewhere (2).

Table A1 The herbal components of *Sheng Mai Yin*

Herbal name	Botanical name	Plant parts used	Chinese name	Quality control marker	Reference to Chinese Pharmacopoeia (Part 1, 2020 Edition)
Ginseng Radix et Rhizoma	*Panax ginseng C.A.Mey.*	Root and rhizome	Hong Shen	Ginsenoside Rg1, Ginsenoside Re	Page 160
Ophiopogonis Radix	*Ophiopogon japonicus (L.f) Ker-Gawl.*	Root	Mai Dong	Ruscogenin	Page 162
Schisandrae Chinensis Fructus	*Schisandra chinensis (Turcz.) Baill.*	Fruit	Wu Wei Zi	Schisandrol A	Page 68

2. Taxonomic authentication

Taxonomic authentication in the Chinese Pharmacopoeia is primarily based on macroscopic/microscopic characteristics, and thin-layer chromatography (TLC). Although DNA

barcoding-based taxonomic authentication is not currently included in the Chinese Pharmacopoeia, such methods are widely accepted and utilized in China for the authentication of botanical drugs.

2.1 *Ginseng Radix et Rhizoma*

Macroscopic Characteristics: The primary root is fusiform or cylindrical, measuring 3–15 cm in length and 1–2 cm in diameter. The surface is grayish-yellow, with sparse, shallow, and discontinuous transverse wrinkles or striations on the upper part or throughout, accompanied by distinct longitudinal furrows. Two to three lateral roots emerge from the lower part, bearing numerous slender fibrous roots, which often exhibit inconspicuous small verrucous protrusions. The rhizome is 1–4 cm long and 0.3–1.5 cm in diameter, often contorted and curved, with adventitious roots and sparsely distributed pit-like stem scars. The texture is relatively firm. The cross-section is pale yellowish-white with a powdery appearance. The cambial ring appears yellowish-brown, and the phloem contains punctate resin canals and radial fissures of yellowish-brown color. It has a distinctive aroma and a slightly bitter and sweet taste. Alternatively, the primary root may be similar in length to or shorter than the rhizome, presenting a cylindrical, prismatic, or forked (the Chinese character "人" shape) form, ranging from 1–6 cm in length. The surface is grayish-yellow with longitudinal wrinkles and ring-like striations on the upper or mid-lower portions. There are typically 2–3 lateral roots with fewer, slender, and neatly arranged fibrous roots that show more prominent verrucous protrusions. The rhizome is slender, though some may be short and thick, with sparse or densely distributed and deeply sunken stem scars on the upper or middle sections. The adventitious roots are thin and mostly hang downward.

Microscopic Characteristics: The transverse section reveals several layers of cork cells. The phelloderm is narrow. Fissures are present in the outer phloem, while the inner phloem consists of densely arranged parenchyma cells, with scattered resin canals containing yellow secretions. The cambium forms a continuous ring. The xylem rays are broad, with vessels appearing singly or in small groups, arranged intermittently in a radial pattern. Occasionally, non-lignified fibers are found adjacent to the vessels. Parenchyma cells contain rosette-like calcium oxalate crystals. The powder is pale yellowish-white. Fragments of resin canals are easily observed, containing yellow, amorphous secretions. Calcium oxalate rosettes range from 20 to 68 μm in diameter, with sharply pointed edges. Cork cells appear polygonal or nearly square in surface view, with slightly undulating walls. Reticulate and scalariform vessels are 10–56 μm in diameter. Starch granules are abundant: simple

granules are spherical, hemispherical, or irregularly polygonal, 4–20 μm in diameter, with punctate or fissured hilum; compound granules are composed of 2 to 6 subgranules.

TLC: Weigh 1 g of the powdered sample and add 40 mL of chloroform. Reflux for 1 hour, then discard the chloroform extract. Evaporate residual solvent from the residue, moisten with 0.5 mL of water, and add 10 mL of water-saturated n-butanol. Sonicate for 30 minutes, then collect the supernatant and mix it with three times its volume of ammonia solution. Shake thoroughly and allow the layers to separate. Collect the upper layer and evaporate to dryness. Dissolve the residue in 1 mL of methanol to obtain the test solution. Prepare a reference herbal material solution by treating 1 g of Panax ginseng reference material in the same manner. Separately, prepare a reference standard solution containing 2 mg/mL each of ginsenosides Rb1, Re, Rf, and Rg1 in methanol. Apply 1–2 μL of each solution to the same silica gel G TLC plate. Use the lower phase of a mixture of chloroform–ethyl acetate–methanol–water (15:40:22:10), previously equilibrated at below 10 °C, as the developing solvent. After development, remove and air-dry the plate, spray with 10% ethanolic sulfuric acid solution, and heat at 105 °C until the spots are clearly visible. Observe under both daylight and UV light at 365 nm. In the chromatogram of the test sample, spots of the same color or fluorescence should appear at the same positions as those in the chromatograms of the reference herbal material and reference standards.

2.2 *Ophiopogonis Radix*

Macroscopic Characteristics: The crude drug is fusiform, with slightly tapered ends, measuring 1.5–3 cm in length and 0.3–0.6 cm in diameter. The surface is pale yellow or grayish-yellow with fine longitudinal striations. It is pliable and tough in texture. The cross-section is yellowish-white, translucent, with a small central stele. It has a slightly aromatic odor and a sweet, slightly bitter taste.

Microscopic Characteristics: In transverse section, the epidermis consists of a single layer of cells, which may be detached. The periderm comprises 3–5 layers of lignified cells. The cortex is broad, containing mucilage cells with bundles of calcium oxalate needle crystals, some up to 10 μm in diameter. Endodermal cell walls are uniformly thickened and lignified, with passage cells. One layer of stone cells is present outside the endodermis; their inner and lateral walls are thickened and densely pitted. The central stele is small, with 16–22 phloem bundles. The xylem forms a ring

consisting of vessels, tracheids, wood fibers, and lignified parenchyma on the inner side. The pith is small, and composed of rounded parenchymatous cells.

TLC: Weigh 2 g of the powdered sample and cut it into small pieces. Add 20 mL of a chloroform–methanol mixture (7:3), soak for 3 hours, and sonicate for 30 minutes. Cool and filter. Evaporate the filtrate to dryness, and dissolve the residue in 0.5 mL of chloroform to obtain the test solution. Prepare a reference herbal material solution using 2 g of Ophiopogon japonicus in the same manner. Apply 6 μL of each solution to the same silica gel GF254 TLC plate. Use a mixture of toluene–methanol–glacial acetic acid (80:5:0.1) as the developing solvent. After development, remove and air-dry the plate. Examine under UV light at 254 nm. In the chromatogram of the test solution, a spot of the same color appears at the same position as that in the reference herbal material chromatogram.

2.3 *Schisandrae Chinensis Fructus*

Macroscopic Characteristics: The crude drug is irregularly spherical or subspherical, with a diameter of 5–8 mm. The surface appears red, purplish-red, or dark red, wrinkled and oily in texture; some may appear blackish-red or display a "frosted" appearance. The flesh is soft. Each fruit contains one or two kidney-shaped seeds, which are yellowish-brown, glossy, and have a thin, brittle seed coat. The fruit has a faint odor and a sour taste; when broken, the seeds emit an aromatic smell and have a pungent, slightly bitter taste.

Microscopic Characteristics: In transverse section, the exocarp is composed of a single layer of square or rectangular cells with slightly thickened walls, covered by a cuticle and scattered with oil cells. The mesocarp contains over ten layers of parenchyma cells with starch granules and scattered small collateral vascular bundles. The endocarp is formed by a single layer of small parenchyma cells. The outermost layer of the seed coat consists of one row of radially elongated sclereids with thick walls and densely distributed pits and fissures. Beneath it are several layers of rounded, triangular, or polygonal sclereids with larger pits. Below the sclereid layers are several rows of parenchyma cells. Vascular bundles are present at the raphe region. The oil cell layer is formed by a row of rectangular cells containing brownish-yellow oil droplets, followed by 3–5 layers of small cells. The innermost epidermis of the seed coat comprises a single layer of small cells with slightly thickened walls. The endosperm cells contain oil droplets and aleurone grains. The powder appears dark purple. Surface-view of sclereids from the seed coat epidermis shows polygonal or elongated polygonal cells (18–50 μm in diameter) with thick walls and extremely fine fissures, containing dark

brown substances. The inner seed coat sclereids are polygonal, subrounded, or irregular in shape, up to 83 μm in diameter, with slightly thickened walls and large pits. Surface-view of pericarp epidermal cells shows polygonal shapes with slightly beaded thickening of the anticlinal walls and cuticular striations; oil cells are scattered throughout. The mesocarp cells are shrunken, containing dark brown substances and starch granules.

TLC: Weigh 1 g of powdered sample and add 20 mL of chloroform. Reflux for 30 minutes, filter, and evaporate the filtrate to dryness. Dissolve the residue in 1 mL of chloroform to obtain the test solution. Prepare a reference herbal material solution using 1 g of Schisandra chinensis by the same procedure. Prepare a reference solution of Schisandrin A by dissolving in chloroform to make a 1 mg/mL solution. According to the method described in General Rule 0502 of the Chinese Pharmacopoeia, spot 2 μL each of the test solution, reference herbal material solution, and reference compound solution on the same silica gel GF254 plate. Develop using the upper phase of a mixture of petroleum ether (30–60 °C)–ethyl acetate–formic acid (15:5:1) as the mobile phase. After development, remove the plate, dry it, and examine under UV light at 254 nm. In the chromatogram of the test solution, a spot of the same color appears at the corresponding positions as in the reference herbal material and reference compound chromatograms.

3. Sourcing

The SMY used in this study is manufactured by Tong Ren Tang Technologies Co. Ltd., a Good Manufacturing Practice (GMP)-certified pharmaceutical company (GMP certificate number: HE20190029M). The herbal product is sourced from the National Agricultural Science and Technology Park, Yutian County, Tangshan City, Hebei Province, China. The batch number for this trial is 24262304.

4. Preparation

The preparation of SMY follows the method described in the Chinese Pharmacopoeia (Part 1, 2020 Edition) (3). Weigh 100 g of *Ginseng Radix et Rhizoma*, 200 g of *Ophiopogonis Radix*, and 100 g of *Schisandrae Chinensis Fructus* and grind them into coarse powder. Soak the mixture in 65% ethanol for 24 hours, then perform percolation. Collect approximately 4500 mL of percolate and concentrate it under reduced pressure to about 250 mL. Cool the concentrate, add 400 mL of purified water to dilute, and filter. Add 300 mL of 60% syrup and an appropriate amount of preservative

(ethylparaben). Adjust the pH to the specified range (4.5–7.0), and add purified water to a final volume of 1000 mL. Mix well, allow to stand, filter again, fill into containers, and sterilize to obtain the final preparation. The final product is a clear, yellowish-brown to reddish-brown liquid with an aromatic odor and a sweet, slightly sour, and slightly bitter taste.

5. Standardization and quality control

According to the Chinese Pharmacopoeia, standardization of SMY is achieved by specifying Schisandrol A as the primary marker, which should be ≥ 0.25 mg per 10 mL vial (3). High-performance liquid chromatography (HPLC) is the official analytical method used for its quantification. In this trial, SMY will be procured from a single GMP-certified manufacturer (Tong Ren Tang Technologies Co., Ltd.; GMP No. HE20190029M), and a single-lot strategy (Batch No. 24262304) will be implemented across all sites to minimize inter-batch heterogeneity whenever feasible. Lot quality control will include HPLC quantification of the pharmacopeial marker Schisandrol A. Retention samples from the release lot will be archived under controlled conditions. If an additional lot becomes necessary, a pre-specified batch-bridging procedure will be undertaken before dispensing, consisting of side-by-side HPLC quantification of Schisandrol A against the retention sample from the original lot. Acceptance will require (i) meeting the pharmacopeial specification (≥ 0.25 mg per 10 mL) and (ii) demonstrating comparability within a predefined ±10% relative difference versus the original lot. Lot numbers will be recorded at receipt and dispensing, if an additional lot is used.

6. Safety assessment

In China, *Ginseng Radix et Rhizoma*, *Ophiopogonis Radix*, and *Schisandrae Chinensis Fructus* have long been used as raw materials in health food products, reflecting their well-established safety profile. Accordingly, no clinical toxicity or adverse events have been reported for SMY (2). Moreover, meta-analyses have demonstrated that SMY exhibits a favorable safety profile in the treatment of cardiovascular diseases, including anthracycline-induced cardiotoxicity (4-7).

7. Regulatory adherence

SMY is a traditional Chinese medicine (TCM) oral compound prescription approved by the National Medical Products Administration (NMPA) of China (Z11020363). The product used in this trial is manufactured by Tong Ren Tang Technologies Co., Ltd., which holds a valid GMP certificate

(HE20190029M). The specific batch used in this study is Batch Number 24262304. None of the herbal components used in SMY are listed as endangered species under CITES or require compliance with the Nagoya Protocol. The manufacturer adheres to all applicable phytosanitary regulations and traceability standards issued by the NMPA of China.

SMY has been approved for clinical use in China for the treatment of *Qi* and *Yin* deficiency (QYD) syndrome, characterized by symptoms such as palpitations, shortness of breath, and spontaneous sweating, and is commonly used in the management of cardiovascular diseases. To date, no clinical toxicity or adverse effects have been reported for SMY in routine clinical use, supporting its favorable safety profile under current regulatory oversight.

References

1. Heinrich M, Jalil B, Abdel-Tawab M, Echeverria J, Kulic Z, McGaw LJ, et al. Best practice in the chemical characterisation of extracts used in pharmacological and toxicological research—the ConPhyMP—guidelines12. Front Pharmacol. (2022) Volume 13 - 2022.
2. Ouyang Y, Tang L, Hu S, Tian G, Dong C, Lai H, et al. Shengmai san-derived compound prescriptions: a review on chemical constituents, pharmacokinetic studies, quality control, and pharmacological properties. Phytomedicine. (2022) 107: 154433. doi: https://doi.org/10.1016/j.phymed.2022.154433
3. Chinese Pharmacopoeia Commission. Pharmacopoeia of the people's republic of china (part 1, 2020 edition). (2020): 835.
4. Zhou Q, Qin WZ, Liu SB, Kwong J, Zhou J, Chen J. Shengmai (a traditional chinese herbal medicine) for heart failure. Cochrane Database Syst Rev. (2014). doi: 10.1002/14651858.CD005052.pub5
5. Li Y, Li D, Jin X, Yang S, Zhao R, Wu M. Efficacy and safety of shengmai preparation combined with western medicine for coronary heart disease: a systematic review and meta-analysis. Am J Chin Med. (2021) 50: 133-59. doi: 10.1142/S0192415X22500057
6. Zhang X, Li Y, Zhang Y, Zhang W, Zhu Y, Zhang J, et al. Shengmai san for treatment of cardiotoxicity from anthracyclines: a systematic review and meta-analysis. Chin J Integr Med. (2022) 28: 374-83. doi: 10.1007/s11655-022-2884-5
7. Zhang B, Liu X, Ling Y, Lu C, Jin X, Wei Y, et al. Effectiveness and safety of shengmai san for viral myocarditis: a systematic review and meta-analysis of randomized controlled trials. Cardiovasc Ther. (2024) 2024: 2127018. doi: https://doi.org/10.1155/2024/2127018
